# Impact of timing and combination of different BNT162b2 and ChAdOx1-S COVID-19 basic and booster vaccinations on humoral immunogenicity and reactogenicity in adults

**DOI:** 10.1038/s41598-023-34961-8

**Published:** 2023-06-03

**Authors:** Simon Dedroogh, Sven Schmiedl, Petra A. Thürmann, Katharina Graf, Sebastian Appelbaum, Reinhard Koß, Christian Theis, Zewarudin Zia, Jürgen Tebbenjohanns, Serge C. Thal, Michael Dedroogh

**Affiliations:** 1 Chair of Anesthesiology I, Department of Medicine, Faculty of Health, Witten/Herdecke University, Witten, Germany; 2 Center for Clinical Trials, Department of Medicine, Faculty of Health, Witten/Herdecke University, Witten, Germany; 3 Chair of Clinical Pharmacology, Department of Medicine, Faculty of Health, Witten/Herdecke University, Witten, Germany; 4 Philipp Klee-Institute for Clinical Pharmacology, Helios University Hospital Wuppertal, Wuppertal, Germany; 5 Department of Psychology and Psychotherapy, Faculty of Health, Witten/Herdecke University, Witten, Germany; 6 Department of Occupational Medicine, Helios Klinikum Hildesheim, Hildesheim, Germany; 7 Department of Anesthesiology, Helios Klinikum Hildesheim, Hildesheim, Germany; 8 Department of Cardiology, Helios Klinikum Hildesheim, Senator-Braun-Allee 33, 31135 Hildesheim, Germany; 9 Department of Anesthesiology, Helios University Hospital Wuppertal, Witten/Herdecke University, Heusnerstrasse 40, 42283 Wuppertal, Germany

**Keywords:** Infection, Infectious diseases, Vaccines, Drug safety, Health care, Medical research, RNA vaccines, SARS-CoV-2

## Abstract

In this single-center observational study with 1,206 participants, we prospectively evaluated SARS-CoV-2-antibodies (anti-S RBD) and vaccine-related adverse drug reactions (ADR) after basic and booster immunization with BNT162b2- and ChAdOx1-S-vaccines in four vaccination protocols: Homologous BNT162b2-schedule with second vaccination at either three or six weeks, homologous ChAdOx1-S-vaccination or heterologous ChAdOx1-S/BNT162b2-schedule, each at 12 weeks. All participants received a BNT162b2 booster. Blood samples for anti-S RBD analysis were obtained multiple times over a period of four weeks to six months after basic vaccination, immediately before, and up to three months after booster vaccination. After basic vaccination, the homologous ChAdOx1-S-group showed the lowest anti-S RBD levels over six months, while the heterologous BNT162b2-ChAdOx1-S-group demonstrated the highest anti-S levels, but failed to reach level of significance compared with the homologous BNT162b2-groups. Antibody levels were higher after an extended vaccination interval with BNT162b2. A BNT162b2 booster increased anti-S-levels 11- to 91-fold in all groups, with the homologous ChAdOx1-S-cohort demonstrated the highest increase in antibody levels. No severe or serious ADR were observed. The findings suggest that a heterologous vaccination schedule or prolonged vaccination interval induces robust humoral immunogenicity with good tolerability. Extending the time to boost-immunization is key to both improving antibody induction and reducing ADR rate.

## Introduction

The emergence of SARS-CoV-2 in 2019 rapidly escalated into a global pandemic, causing significant morbidity and mortality during the acute phase. More recently, focus has shifted towards understanding the long-term effects of SARS-CoV-2 infection, referred to as post-COVID-19 and long-COVID-19 syndromes^1–3^. The authorized vaccines represent a promising strategy to mitigate both acute and long-term COVID-19 syndromes. Despite this, considerable skepticism regarding their safety and side-effect profiles persists, attributable in part to the innovative mRNA technologies and accelerated approval processes.

To rapidly achieve widespread immunization within the European Union (EU) population, national authorities have proposed various combinations of vaccines, as well as modifications in timing and dosing. Nonetheless, there is limited evidence on how specific combinations and revaccination schedules impact immune response and side-effects^4–6^ Optimization of vaccination regimens, through judicious vaccine selection and well-timed dosing intervals, has the potential to augment both individual and population-level immunity, thereby contributing to superior public health outcomes. In the endeavor to mitigate the prevalence of post-acute sequelae of SARS-CoV-2 infection, commonly known as long COVID, vaccination has emerged as a critical instrument. Recent literature indicates that individuals who had received a minimum of one dose of any of the three authorized COVID-19 vaccines prior to contracting the virus exhibited a reduced likelihood of experiencing long COVID symptoms compared to their unvaccinated counterparts^7^.

To address this knowledge gap, the Helios Hildesheim COVID-19 Vaccination Study (HelCo-Vac) was designed to prospectively investigate the reactogenicity and immunogenicity of homologous and heterologous vaccine combinations. This study focuses on the generation of SARS-CoV-2 anti-spike antibodies in healthcare workers following basic and booster vaccinations, with a particular emphasis on the BNT162b2 and ChAdOx1-S vaccines.

## Results

### Demographics and study population

A total of 1,338 clinical staff members received their initial vaccination (BNT162b2: n = 565 [42.2%]; ChAdOx1-S: n = 773 [57.8%]) and underwent eligibility assessment for study participation. Two individuals were excluded due to prior SARS-CoV-2 infection. Ultimately, 1,206 participants (90.1% of the vaccinated population) provided informed consent and were enrolled in the study.

The study cohort was stratified into four distinct groups based on the timing of vaccination and the specific vaccination protocol employed. Table 1 delineates the primary demographic characteristics of each group, as well as the corresponding vaccination intervals.

**Table 1:** Baseline characteristics of the study cohorts at the time of the second vaccination, N = 1118.

Characteristics	Study-cohorts after second vaccination			
	BB-3 N = 248	BB-6 N = 262	AA-12 N = 265	AB-12 N = 343
Interval between first to second vaccination—weeks	3	6	12	12
Median interval between second to booster—vaccination (range)—weeks	42 (33–46)	30 (24–32)	26 (22–31)	26 (22–29)
Median age	46	46	52	46
(IQR; range)—years	(35–53.5; 19–64)	(32–56; 19–73)	(39.5–59; 21–70)	(34.5–54; 18–71)
Sex—n (%)^a^				
Male	93 (38)	50 (19.5)	59 (22.7)	55 (16.8)
Female	152 (62)	207 (80.5)	201 (77.3)	273 (83.2)
Median BMI	25.2	24.8	24.9	24.9
(IQR; range)—kg/m^2^	(22.9–29.1; 19–43)	(22.3–28.6; 16–52)	(22.3–28.7; 17–50)	(22.4–28.8; 16–46)
Occupational group—n (%)^a^				
Nursing	129 (53.3)	40 (17.6)	120 (50.2)	105 (37.1)
Physicians	86 (35.5)	32 (14.1)	13 (5.4)	37 (13.1)
Other hospital professions	27 (11.2)	155 (68.3)	106 (44.4)	141 (49.8)

Baseline immunizations (first and second vaccination) were performed with m-RNA vaccine BNT162b2 (BioNTech/Pfizer, Mainz, Germany; B) and vector-based vaccine ChAdOx1-S (AstraZeneca, Wilmington, DE; A). In each case, cohort designations are composed of the first and second vaccines (A and B, respectively) and the interval between first and second vaccinations (in weeks).

^a^Numerical differences between individual characteristics and the total population are due to missing information in the questionnaire.

The study population's age ranged from 18 to 71 years, with a median age of 47 years (IQR 35–55), representing a wide spectrum of the working population. Nurses constituted the largest employee group at the hospital, resulting in a predominantly female study population (female n = 833 [76.4%]). Differences in age and job categories across the four study cohorts mirrored the evolving priority recommendations of the German Standing Committee on Vaccination (STIKO) during the study period.

Post-second vaccination, 1118 participants remained in the study, while 485 subjects persisted after the booster vaccination (see Supplement eTable 3, eFigs. 1, 2 for additional details).

### Influence of vaccination protocols on anti-S RBD titers formation

**Figure 1:**
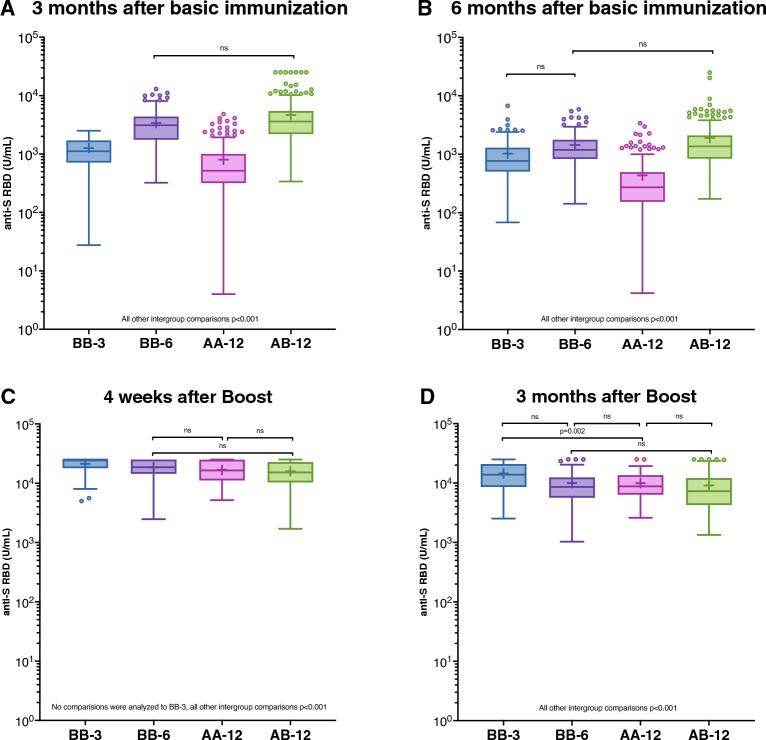
Anti-S RBD levels between the investigated vaccination groups at different time points after basic immunization and booster vaccination. Data are presented in the standard box plot format: The box ranges from the first quartile (Q1) to the third quartile (Q3) of the distribution and the range represents the interquartile range (IQR). The median is represented by a line across the box, the mean is depicted by a "+" in the box. Whiskers range from Q1 and Q3 to 1.5 times IQR. Data beyond the whiskers are shown as outliers. Only vaccination cohorts with a median anti-S RBD concentration below the upper limit of quantification (ULoQ) were considered for statistical analysis using the Wilcoxon test.

The primary endpoint of the study involved measuring anti-S RBD levels following various vaccination protocols (Fig. 1). After the initial immunization (Fig. 1A,B), the highest median anti-S RBD levels were observed at six months in the BB-6 (median 1188 U/mL [IQR 830–1766]) and AB-12 (1362 U/mL [842–2103]) groups. The BB-3 group exhibited significantly lower antibody levels at four weeks and three months compared to BB-6 but did not reach level of significance at six months (769 U/mL [509–1296]). The AA-12 group demonstrated a significantly lower median anti-S RBD level (272 U/mL [154–488]; all intergroup comparisons p < 0.001 [Wilcoxon test]). Consequently, a homologous vaccination protocol using ChAdOx1-S was deemed inferior to homologous vaccination with BNT162b2 and a heterologous protocol with ChAdOx1-S and BNT162b2.

Following booster vaccination (Fig. 1C,D), the anti-S RBD levels across groups converged. The AA-12 group, with a median of 16,488 U/mL (11,192–24,785) at four weeks after booster, caught up with other groups in terms of antibody formation after receiving a heterologous booster. Only the BB-3 group, with a median of 25,000 U/mL (18,474–25,000) at four weeks after booster, displayed a slight advantage over some other groups up to three months after booster: BB-3 vs BB-6 (18,670 U/mL [14,428–25,000]) four weeks after booster and BB-3 vs AA-12 three months after booster; each p = 0.002; BB-3 vs BB-6 three months after booster p = 0.003; BB-3 vs AB-12 at four weeks after booster (15,171 U/mL [10,393–22,783]) and all other group comparisons to BB-3 p < 0.001 (Wilcoxon test, adjusted α = 0.002).

It is important to note that subjects in the BB-3 cohort experienced a longer waiting period between completing the initial and booster vaccination compared to the other groups (median 42 weeks [range 33–46]; Table 1).

Given the study population's female predominance (refer to Table 1) in our non-randomized observational study, this could be a confounding factor in the event of an unequal sex distribution across the four study cohorts. Therefore, additional analyses were conducted to demonstrate the influence of sex on the study outcome “concentration of anti-S RBD”. Detailed results are provided in the supplement (eTables 7–9). However, the relevance of sex on the reported outcomes was largely ruled out.

### Influence of basic immunization and booster on anti-S RBD kinetics

**Figure 2:**
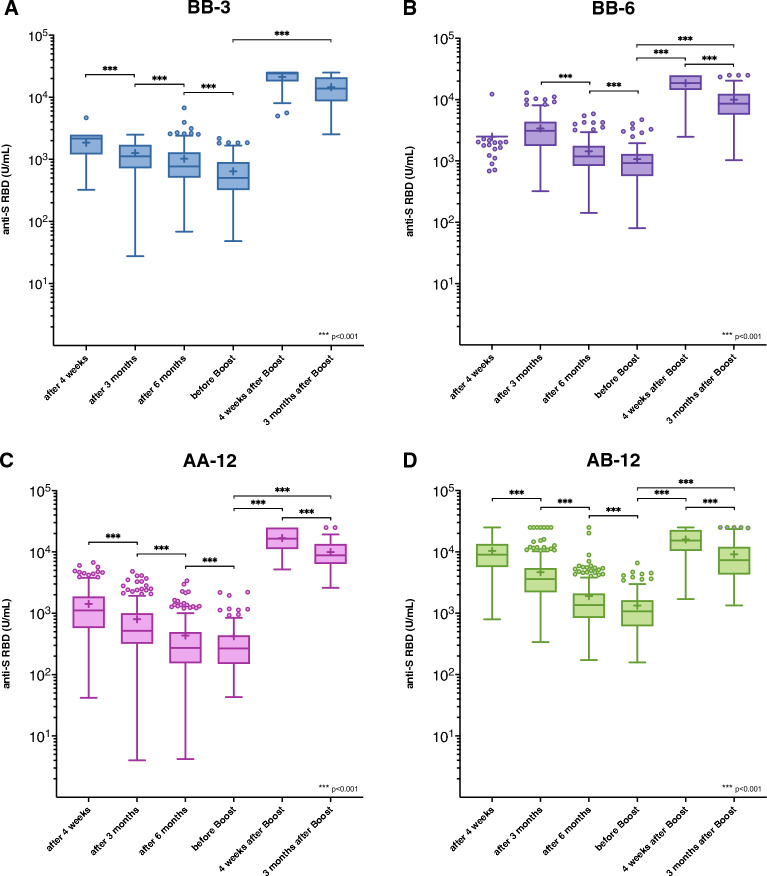
Anti-S RBD levels within the investigated vaccination groups at different time points after basic immunization and booster vaccination. Data are presented in the standard box plot format: The box ranges from the first quartile (Q1) to the third quartile (Q3) of the distribution and the range represents the interquartile range (IQR). The median is represented by a line across the box, the mean is depicted by a “+” in the box. Whiskers range from Q1 and Q3 to 1.5 times IQR. Data beyond the whiskers are shown as outliers. Only study visits with a median anti-S RBD concentration below the upper limit of quantification (ULoQ) were considered for statistical analysis using the Wilcoxon signed rank test.

The study´s secondary endpoint aimed to evaluate the change in anti-S RBD levels over time after basic immunization and booster in all vaccination groups (Fig. 2A–D). As expected, anti-S RBD levels in all groups significantly declined over time (six months after basic immunization (BB-3: median 769 U/mL [IQR 509–1296]; BB-6: 1,188 U/mL [830–1766]; AA-12: 272 U/mL [154–488]; AB-12: 1362 U/mL [842–2103]) compared to immediately before booster (BB-3: 501 U/mL [326–900]; BB-6: 927 U/mL [576–1297]; AA-12: 268 U/mL [152–429]; AB-12: 1073 U/mL [640–1626]; all intragroup comparisons p < 0.001 [Wilcoxon signed rank test]).

After booster vaccination, median anti-S RBD levels significantly increased in all groups from immediately prior boost to four weeks after (BB-3: 25,000 U/mL [18,474–25,000]; BB-6: 18,670 U/mL [14,428–25,000]; AA-12: 16,488 U/mL [11,192–24,785]; AB-12: 15,171 U/mL [10,393–22,783]; p < 0.001 [Wilcoxon signed rank test]).

### Effect of booster vaccination on anti-S RBD level

**Figure 3:**
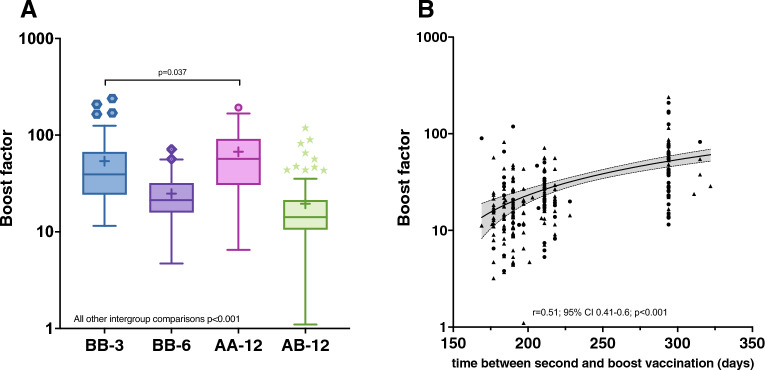
Effect of booster vaccination on anti-S RBD kinetics (boost-factor: quotient of anti-S RBD levels four weeks after and levels immediately before the booster vaccination). (**A**) Booster effect per vaccination-cohort. Data are presented in the standard box plot format: The box ranges from the first quartile (Q1) to the third quartile (Q3) of the distribution and the range represents the interquartile range (IQR). The median is represented by a line across the box, the mean is depicted by a "+" in the box. Whiskers range from Q1 and Q3 to 1.5 times IQR. Data beyond the whiskers are shown as outliers. The statistical analysis was performed using the Wilcoxon test. (**B**) Correlation between booster factor and time-interval (in days) between second and booster vaccination, without considering the homologous ChAdOx1-S vaccination group, which was cross-vaccinated with m-RNA vaccine for the first time. Each symbol represents an individual subject: Subjects marked with a circle showed an antibody measurement value above the upper limit of quantification (ULoQ) four weeks after the boost. Subjects marked with a triangle showed only continuous antibody values. As a statistical test, a Spearman Correlation was used.

The impact of the booster vaccination on anti-S RBD antibody levels was investigated separately in a post hoc analysis, and a "boost factor" was calculated as quotient of anti-S RBD levels four weeks after and levels immediately before the booster vaccination (Fig. 3, Panel A). Both, BB-3 (median 39 [IQR 24–67]) and AA-12 (median 57 [IQR 31–91]) showed a significantly stronger increase in anti-S antibody levels compared to the other two groups: BB-6 (median 21 [IQR 16–32]); AB-12 (median 14 [IQR 11–21]); BB-3 vs AA-12 p = 0.037 [Wilcoxon test]; all other comparisons p < 0.001 [Wilcoxon test]).

To investigate the impact of waiting time between the repeated vaccination, each individual subject's boost factor was correlated with the time interval (in days) between the second and third vaccination. The analysis revealed a significant influence of the interval on the increase of blood antibodies after booster vaccination (Spearman correlation r = 0.51; 95% CI 0.41–0.6; p < 0.001).

Subjects of group AA-12 were excluded from this analysis since they were cross-vaccinated with BNT162b2 for the first time.

The key statements remain valid even when considering only continuous antibody values for the analysis, excluding values above the ULoQ. Additional results are provided in the Supplement (eFig. 3).

### Reactogenicity: influence of protocols on adverse reactions

**Figure 4:**
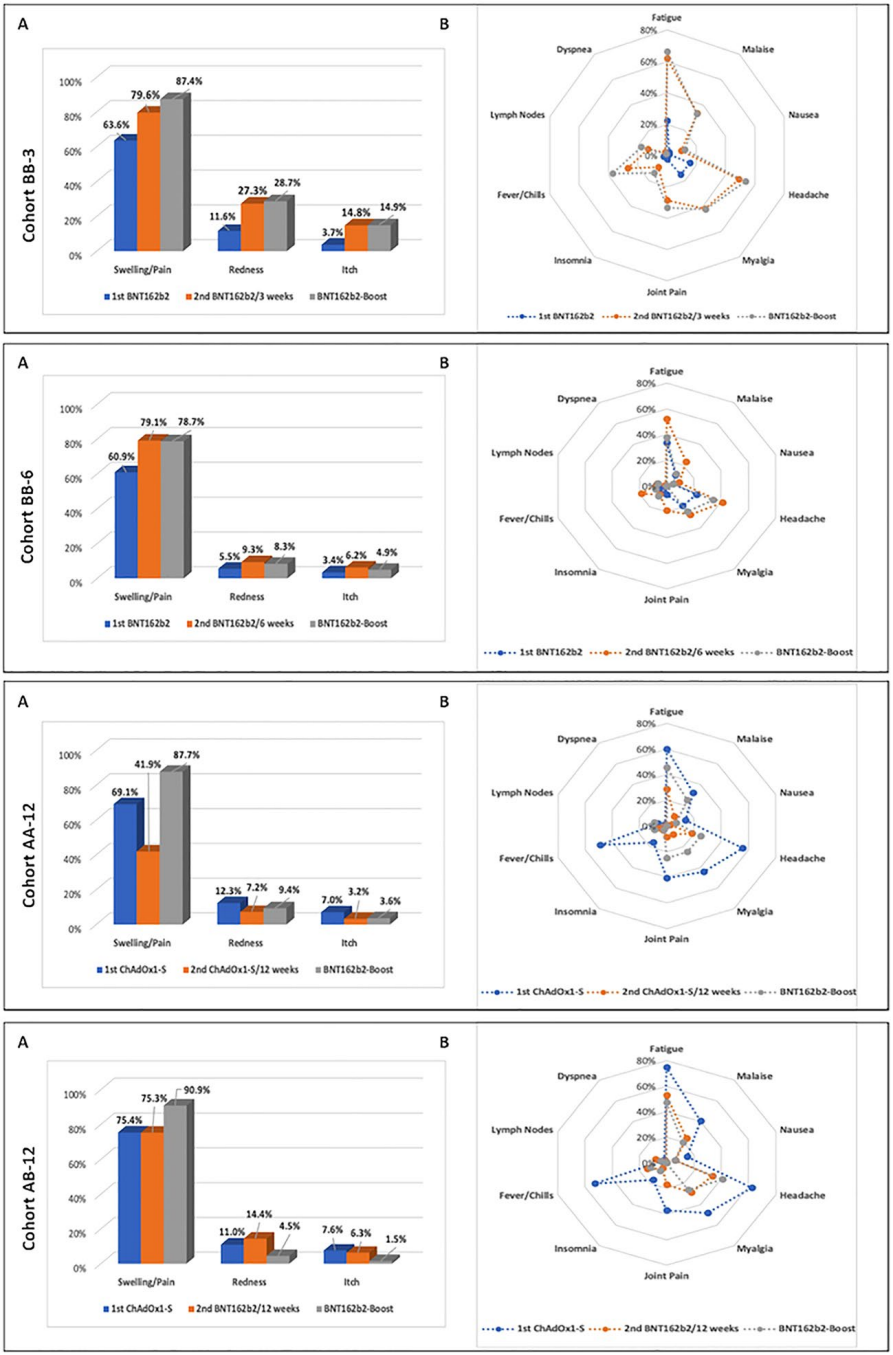
Reactogenicity. Frequencies (in %) and distributions of local (**A**) and systemic (**B**) adverse drug reactions (ADR) within seven days after first/second and booster vaccination per cohort.

In this study, the frequencies and distributions of local and systemic adverse drug reactions (ADR) were prospectively recorded for seven days after vaccination at all three vaccinations (Fig. 4, eTable 4). The study participants reported a typical distribution pattern of ADR, consisting of local swelling/pain, fatigue, headache, myalgia, joint pain, and fever/chills. This pattern was consistently reported with all vaccine combinations and number of vaccinations.

After the first vaccination, ChAdOx1-S proved to be clinically more reactogenic than BNT162b2. Any ADR was reported more frequently in both ChAdOx1-S-groups AA-12 (216/241; 89.6%) and AB-12 (274/288; 95.1%) compared to the BNT162b2-groups BB-3 (173/236; 73.3%) and BB-6 (172/235; 73.2%; each p < 0.001 [Chi^2^ test]). The rate of local and systematic ADR and their severities was similar in the following immunizations (eTables 4 and 5). Almost one in two subjects reported fever and chills after ChAdOx1-S primary vaccination (268/533; 50.3%). Cohorts using the same first vaccine did not report differences in the severity of ADR (BB-3 vs BB-6: p = 0.321 [Chi^2^ test]; AA-12 vs AB-12: p = 0.138 [Chi^2^ test]; eTable 5).

After the second dose, AA-12 showed the lowest ADR rate (no ADR reported: AA-12 (97/220; 44.1%) vs BB-3 (14/211; 6.6%) and BB-6 (33/194; 17.0%) and AB-12 (35/268; 13.1%): each comparison p < 0.001 [Chi^2^ test]). Homologous BNT162b2 vaccination with a three-week interval showed the most pronounced reactogenicity (BB-3 vs AB-12: p = 0.021 [Chi^2^ test]; BB-3 vs BB-6 p = 0.007 [Chi^2^ test]). In this respect, the subjects with a three-week extension of the immunization interval tolerated the BNT162b2 vaccine significantly better. In comparison, a heterologous vaccination (AB-12) was tolerated as well as homologous BNT162b2 vaccination with an extended vaccination interval (p = 0.448 [Chi^2^ test]).

The ADR of the boost vaccination was clinically similar in BB-3, BB-6, and AB-12, whereas AA-12, which was cross-vaccinated with BNT162b2 for the first time, exhibited higher reactogenicity (53/57; 93%) compared with the second vaccination (123/220; 55.9%; eTables 4 and 5).

In line with the reported ADR, the rate of incapacity to work in the first weeks after immunization was also dependent on the protocol (eTable 6). After the first immunization, significantly more subjects reported inability to work with ChAdOx1-S (14.4%) compared to BNT162b2 (0.9%). Interestingly, the second repeated vaccination was much better tolerated with ChAdOx1-S compared to BNT162b2 (BB-3: 6.9%, BB-6: 4.1% vs AA-12: 1.4%; p < 0.05).

## Discussion

The study presented here is the first to examine the effects of vaccine combinations and timing of redosing on blood antibody response and the incidence of adverse drug reactions (ADR) in a group of 1,206 medical health care professionals trained to identify such symptoms. Our key findings indicate that prolonging the interval between the two basic immunizations or between the second and booster vaccination leads to an increase in the antibody response. Interestingly, delaying the second BNT162b2 vaccination results in high levels of blood antibodies while keeping the rate of ADR low. In contrast, ChAdOx1-S leads to high levels of ADRs after the first but not the second immunization.

Previous research has shown the importance of the vaccination interval between natural infection and booster vaccination. In these studies, as in our results, a longer vaccination interval correlated directly with increased immunogenicity. One possible explanation is that the immune response of the spike-specific B cells is impeded by a temporally rapid boost^8^. A similar mechanism could also apply to the time interval between two vaccinations.

Our ADR data also suggest that incapacity to work is less frequently observed after the first BNT162b2 and second ChAdOx1-S immunization. Furthermore, delaying the second BNT162b2 from three to six weeks also reduces the number of participants who are unfit to work. These findings clearly demonstrate that the timing of re-immunization is crucial for improving antibody induction and reducing the rate of ADRs.

All investigated vaccine combinations induced a robust increase in spike-specific antibodies after the second vaccination, with the ChAdOx1-S–BNT162b2 heterologous vaccination regime leading to particularly high antibody levels in the study population. Consistent with previous findings, this combination exhibited high antibody blood levels over time compared to the homologous vaccination combinations, while the lowest antibody response was present with the homologous ChAdOx1-S immunization^9–11^. One possible explanation for this phenomenon, which is also supported by animal studies, is that a heterologous vaccination regimen stimulates the immune system more broadly: While mRNA vaccines mainly induce the production of neutralizing antibodies, vector-based vaccines stimulate the production of polyfunctional antibodies^12,13^.

A previous study with the vector based vaccine Ad26.COV2.S also showed low anti-spike SARS-CoV-2 antibody levels compared to mRNA vaccines^14^. An effective measure to increase blood antibody levels was extending the BNT162b2 time interval from three to six weeks, which significantly increased antibody levels at four weeks and three months after basic vaccination in the present study and also in a report from England^15^. Therefore, vaccine combination and time interval are important parameters to achieve an optimal humoral immune response.

Numerous studies have shown that primary vaccination with, for example, BNT162b2 is effective in preventing severe SARS-CoV-2 infections^16–18^. A good correlation of neutralizing antibody levels with total anti-S antibody levels was recently demonstrated^14^, suggesting that high anti-S RBD antibody levels result in a good humoral immune response against SARS-CoV-2. Unfortunately, there is currently no established anti-S RBD antibody threshold that reliably protects against SARS-CoV-2 infection. Additionally, anti-S RBD antibody levels drop markedly over time after primary immunization, independent of the vaccine protocol, and the risk of infection returns^19,20^. Booster vaccination appears to be appropriate to reestablish high antibody levels, which seems even more important in view of the evolving new virus variants, such as B.1.1.529 (Omicron)^21–23^. Recent data show that a booster is important to achieve and maintain high anti-S RBD antibody levels^24,25^. However, little is known about whether the effectiveness of mRNA booster vaccination is dependent on the basic vaccine protocol.

In the present study, a BNT162b2 booster increased anti-S RBD antibody levels in all groups by 10- to almost 100-fold. In fact, antibody levels were markedly higher compared to after basic immunization. This effect appears to be even more important in the groups with homologous ChAdOx1-S vaccination, which show the lowest antibody levels prior to booster immunization, a result which was recently also shown for Ad26.COV2.S basic immunization^14^.

The reactogenicity of the newly developed SARS-CoV-2 vaccines is one of the key factors influencing the acceptance by the population. Due to the similarities between the new and the tested mRNA vaccines, we propose that the data of the present study could help to design an optimized vaccination protocol, especially in case of heterologous vaccine regimes.

In our study, we did not observe any serious or severe adverse drug reactions. Subjects with a heterologous vaccination regimen or an extended vaccination interval showed good vaccine-mediated immunogenicity and at the same time a good tolerability, which is also supported by other data^9^. The BNT162b2 booster as a third vaccination was similarly well tolerated as a second BNT162b2 vaccination. Only the first booster cross-vaccination after a homologous vector-based basic vaccination with ChAdOx1-S showed more ADR compared to a mixed protocol. Incapacity to work, an important socio-economic parameter, increased after the first vaccination, especially in ChAdOx1-S groups and dropped to low levels after second and boost vaccination^26^.

## Limitations

The World Health Organization (WHO) continuously monitors the development of new Variants of Interest and Variants of Concern (VOCs). In Germany, the Omicron variant (B.1.1.529) is currently the dominant variant, surpassing the Alpha (B.1.1.7) or Delta (B.1.617.2) variants. Additionally, sub-variants of Omicron (BA.4 and BA.5) have already been identified in large numbers^27^. The HelCo-Vac-study was conducted at time when B.1.1.7 were prevalent and B.1.617.2 was becoming dominant. The basic vaccination protocols used are less effective against B.1.1.529, and a boost with BNT162b2 several months after the initial immunization can significantly improve efficacy^28,29^. New Omicron-adapted bivalent mRNA booster vaccines are now available and recommended by the National Vaccination Commission in e.g. Germany as a booster vaccination for persons aged 12 years and older. At present, a fourth vaccination with the bivalent BA.5 booster shows no significant advantage in the formation of neutralizing antibodies at four weeks after the booster compared to the monovalent vaccine (WT.1/3)^30^.

Due to the nature of our study, subjects were not randomized into the four vaccination cohorts, resulting in differences in group composition, such as sex and profession. Vulnerable groups, such as the chronically severely ill, immunosuppressed, or particularly elderly were not included, and the male sex is underrepresented. Although other research groups have shown a more distinct immunogenicity and reactogenicity after SARS-CoV-2 vaccination in women^31–33^, we could not fully confirm this with our results. The analyses in our cohort of patients show only to a limited extent a stronger antibody induction in women. However, this does not change the key statements of our study.

During the booster phase of the study, the loss of subjects was due to the availability of several alternative vaccination options outside the study, which were taken up by clinic staff. As a result, systematic follow-up of these subjects was not possible.

Due to changes in instruction of the anti-S RBD level assay by the manufacturer, the upper limit of quantification changed during course of the study. This could have an impact on the analysis of the first samples shortly after the first primary immunization and directly after boost. To address the potential impact of the upper limit of quantification (ULoQ) on anti-S RBD measurements, median-based statistical tests were deliberately used. This approach helps to minimize the potential bias from ULoQ values, thus allowing for a valid interpretation of the results. Analogous methods were used in comparable studies^34^.

Finally, the number of subjects in our study is not sufficient to detect rare or very rare adverse drug reactions. Meta-analyses indicate that severe reactions are a possible result of vaccination against SARS-CoV-2^35^, and they may be missed due to the number of study subjects. HelCo-Vac only investigated vaccine-mediated reactogenicity within one week of vaccination. ADRs occurring later or lasting longer than one week were not recorded and, therefore, outside the study´s scope.

## Conclusion

The present study analyzed antibody levels (anti-S RBD) and clinical parameters in 1,206 medical health care professionals, and identified significant differences in blood antibody response and adverse drug reaction (ADR) rates based on the vaccine combination and timing of the redosing. The ADRs were recorded by self-reporting of trained staff, which suggest a high ADR data quality.

Overall, the data highlights that the timing of re-immunization is crucial in improving antibody induction and reducing the ADR rate. This study provides valuable insights into optimizing vaccination protocols, particularly in the case of heterologous vaccine regimens. By considering the combination of vaccines and the timing of redosing, health care professionals and policymakers can make better-informed decisions to maximize vaccine efficacy and minimize adverse reactions, ultimately improving public health outcomes in the ongoing fight against SARS-CoV-2 and its variants.

## Methods

### Study overview

The HelCo-Vac is a prospective, single-center observational study investigating reactogenicity and immunogenicity following primary immunization and booster vaccination. Hospital staff members were included in the study, with the exception of individuals aged below 18 years and those with known previous SARS-CoV-2 infection. The study received approval by the Ethics Committee of Witten/Herdecke University (No. 52-2021), and all participants provided written informed consent before enrolling in the study. The study was registered at clinicaltrials.gov (NCT 05076227), approved by the German Paul-Ehrlich-Institute (NIS 640), and conducted in accordance with the Declaration of Helsinki^36^. This study was supported by a grant of Helios Kliniken GmbH, Berlin, Germany (ID 2021-0037).

### Study cohorts

The basic immunizations (first and second vaccination) were performed from January to June 2021 using the m-RNA vaccine BNT162b2 (BioNTech/Pfizer, Mainz, Germany; B) and the vector-based vaccine ChAdOx1-S (AstraZeneca, Wilmington, DE; A).

Following the recommendations of the German Standing Committee on Vaccination (STIKO), participants with first dose BNT162b2 received a second BNT162b2 dose either after three or six weeks. Participants with ChAdOx1-S received either ChAdOx1-S or BNT162b2 after 12 weeks at their own discretion. This resulted in three homologous (BB-3 weeks, BB-6 weeks, and AA-12 weeks) vaccine applications and one heterologous BNT162b2-ChAdOx1-S group (AB-12 weeks). BNT162b2 was used to boost all study participants. The time intervals between the second and booster vaccinations are depicted in Table 1.

### Intervention: immunogenicity

Blood samples were obtained at six time points: four weeks, three and six months after completion of the basic immunization, immediately before, four weeks and three months after booster vaccination.

Antibodies against the SARS-CoV-2 receptor-binding domain of the spike protein (anti-S RBD) were quantified for analysis of vaccine-mediated immunogenicity using Elecsys® Anti-SARS-CoV-2 S (Roche, Indianapolis, IN, USA). The lower limit of detection (LLoD) was 0.8 units per milliliter (U/mL) and the upper limit of quantification (ULoQ) was 2,500 U/mL at the beginning of the study and later 25,000 U/mL due to changes in the instructions by the manufacturer (for details see eTable 1 in the Supplement).

Anti-nucleocapsid antibodies (anti-N) were quantified using a double antigen sandwich ELISA Elecsys® Anti-SARS-CoV-2 (Roche) to detect a previous SARS-CoV-2 infection.

### Intervention: reactogenicity

Reactogenicity after first, second, and booster vaccination was assessed using questionnaires on vaccine-induced adverse drug reactions (ADR) within seven days after the respective vaccinations.

The ADR were categorized as three local and ten systemic pre-specified types (local injection-site ADR: swelling/pain, redness, itch; systemic ADR: fatigue, malaise, nausea, headache, myalgia, joint pain, insomnia, fever/chills, lymph node enlargement, dyspnea) and the option “others”. Participants indicated the severity of their ADR on a 1–4 scale (1 = mild symptoms, not affecting daily activities; 2 = moderate symptoms, affecting daily activities; 3 = severe symptoms, preventing daily activities and 4 = severe symptoms, requiring admission to an emergency department). Additionally, incapacity to work was recorded in the first week after vaccination.

### Statistics

Continuous data are presented descriptively by median, quartiles, minimum, and maximum. Values above the upper limit of quantification (ULoQ) were set to the limit. The median is a valid statistic for the location of the distribution if the percentage of ULoQ values is below 50%. Statistical testing for group differences thus were performed with the Wilcoxon test for two groups. For within group comparisons of two time-points Wilcoxon signed rank test was applied.

Categorical variables are described with absolute frequencies and corresponding percentages. Cross-tabulations, Fisher's exact test or the Chi^2^ test were used to inductively describe and compare categorical data. A Spearman correlation was performed to describe the relationship between the booster effect and the time interval between second and third vaccination. To account for multiple testing, Bonferroni correction was applied to evaluate the primary endpoint. Level of significance was set at α = 0.05. Due to the nature of the study, we did not perform an a priori sample size calculation. Data were analyzed using SPSS Statistics version 26 (IBM, Armonk, NY, USA) and R version 4.2.0 (R Core Team).

## Supplementary Information

Supplementary Information.

## Data Availability

The datasets generated during and/or analyzed during the current study are available from the corresponding authors on reasonable request.

## References

[1] Ciaccio M (2021). COVID-19 and Alzheimer’s disease. Brain Sci..

[2] Olliaro PL (2021). An integrated understanding of long-term sequelae after acute COVID-19. Lancet Respir. Med..

[3] Silva Andrade B (2021). Long-COVID and post-COVID health complications: An up-to-date review on clinical conditions and their possible molecular mechanisms. Viruses.

[4] Hillus D (2021). Safety, reactogenicity, and immunogenicity of homologous and heterologous prime-boost immunisation with ChAdOx1 nCoV-19 and BNT162b2: a prospective cohort study. Lancet Respir. Med..

[5] Hyun J (2022). Reactogenicity and Immunogenicity of the ChAdOx1 nCOV-19 coronavirus disease 2019 vaccine in South Korean healthcare workers. Yonsei Med. J..

[6] Kim D-I (2022). Immunogenicity and durability of antibody responses to homologous and heterologous vaccinations with BNT162b2 and ChAdOx1 vaccines for COVID-19. Vaccines.

[7] Simon MA, Luginbuhl RD, Parker R (2021). Reduced incidence of long-COVID symptoms related to administration of COVID-19 vaccines both before COVID-19 diagnosis and Up to 12 weeks after. MedRxiv.

[8] Buckner CM (2022). Interval between prior SARS-CoV-2 infection and booster vaccination impacts magnitude and quality of antibody and B cell responses. Cell.

[9] Nguyen TT (2022). Reactogenicity and immunogenicity of heterologous prime-boost immunization with COVID-19 vaccine. Biomed. Pharmacother..

[10] Sapkota B (2022). Heterologous prime–boost strategies for COVID-19 vaccines. J. Trav. Med..

[11] Chiu N-C (2021). To mix or not to mix? A rapid systematic review of heterologous prime–boost covid-19 vaccination. Expert Rev. Vaccines.

[12] Sadarangani M, Marchant A, Kollmann TR (2021). Immunological mechanisms of vaccine-induced protection against COVID-19 in humans. Nat. Rev. Immunol..

[13] Barrett JR (2021). Phase 1/2 trial of SARS-CoV-2 vaccine ChAdOx1 nCoV-19 with a booster dose induces multifunctional antibody responses. Nat. Med..

[14] Sablerolles RSG (2022). Immunogenicity and reactogenicity of vaccine boosters after Ad26COV2S priming. N. Engl. J. Med..

[15] Amirthalingam G (2021). Serological responses and vaccine effectiveness for extended COVID-19 vaccine schedules in England. Nat. Commun..

[16] Tartof SY (2021). Effectiveness of mRNA BNT162b2 COVID-19 vaccine up to 6 months in a large integrated health system in the USA: A retrospective cohort study. Lancet.

[17] Chemaitelly H (2021). Waning of BNT162b2 vaccine protection against SARS-CoV-2 infection in Qatar. N. Engl. J. Med..

[18] Rosenberg ES (2021). New COVID-19 cases and hospitalizations among adults, by vaccination status—New York, May 3–July 25, 2021. MMWR Morb. Mortal. Wkly. Rep..

[19] Shrotri M (2021). Spike-antibody waning after second dose of BNT162b2 or ChAdOx1. Lancet.

[20] Mizrahi B (2021). Correlation of SARS-CoV-2-breakthrough infections to time-from-vaccine. Nat. Commun..

[21] Lopez Bernal J (2021). Effectiveness of covid-19 vaccines against the B.1.617.2 (delta) variant. N. Engl. J. Med..

[22] Goldberg Y (2021). Waning immunity after the BNT162b2 vaccine in Israel. N. Engl. J. Med..

[23] Teruel, N., Crown, M., Bashton, M. & Najmanovich, R. Computational analysis of the effect of SARS-CoV-2 variant Omicron Spike protein mutations on dynamics, ACE2 binding and propensity for immune escape. 10.1101/2021.12.14.472622 (2021).

[24] Shekhar R, Garg I, Pal S, Kottewar S, Sheikh AB (2021). COVID-19 vaccine booster: To boost or not to boost. Infect. Dis. Rep..

[25] Lustig, Y. *et al.* Superior immunogenicity and effectiveness of the 3rd BNT162b2 vaccine dose. 10.1101/2021.12.19.21268037 (2021).

[26] Nachtigall I (2021). Sex differences in clinical course and intensive care unit admission in a national cohort of hospitalized patients with COVID-19. JCM.

[27] Tallei TE (2023). Update on the omicron sub-variants BA.4 and BA.5. Rev. Med. Virol..

[28] Collie S, Champion J, Moultrie H, Bekker L-G, Gray G (2022). Effectiveness of BNT162b2 vaccine against omicron variant in South Africa. N. Engl. J. Med..

[29] Barda N (2021). Effectiveness of a third dose of the BNT162b2 mRNA COVID-19 vaccine for preventing severe outcomes in Israel: An observational study. Lancet.

[30] Wang Q (2022). Antibody responses to Omicron BA.4/BA.5 bivalent mRNA vaccine booster shot. Biorxiv.

[31] Fischinger S, Boudreau CM, Butler AL, Streeck H, Alter G (2019). Sex differences in vaccine-induced humoral immunity. Semin. Immunopathol..

[32] Klein SL, Marriott I, Fish EN (2015). Sex-based differences in immune function and responses to vaccination. Trans. R. Soc. Trop. Med. Hyg..

[33] Oertelt-Prigione S (2012). The influence of sex and gender on the immune response. Autoimmun. Rev..

[34] Newman J (2022). Neutralizing antibody activity against 21 SARS-CoV-2 variants in older adults vaccinated with BNT162b2. Nat. Microbiol..

[35] Efficacy and safety of COVID-19 vaccines: A systematic review. *Zhongguo Dang Dai Er Ke Za Zhi* **23**, 221–228 (2021). 10.7499/j.issn.1008-8830.2101133 PMC7969187 33691913

[36] World Medical Association Declaration of Helsinki (2013). Ethical principles for medical research involving human subjects. JAMA.

