# Supplementary material for: Impact of timing and combination of different BNT162b2 and ChAdOx1-S COVID-19 basic and booster vaccinations on humoral immunogenicity and reactogenicity in adults
Source: Sci Rep. 2023 Jun 3;13:9036. doi: 10.1038/s41598-023-34961-8 (PMC10239043; doi:10.1038/s41598-023-34961-8)
Supplement: Supplementary file 1 — Supplementary Information. [file 41598_2023_34961_MOESM1_ESM.docx]

**Impact of timing and combination of different BNT162b2 and ChAdOx1-S**

**COVID-19 basic and booster vaccinations on humoral immunogenicity and reactogenicity in adults**

**Supplementary Information**

| **Study Cohorts** | |
| --- | --- |
| **eTable 1.** | Descriptive Statistics of anti-Spike Receptor Binding Domain (anti-S RBD) and Representation of Upper Limits of Quantification (ULoQ) at the different Study Visits. |
| **eTable 2.** | Subjects in the respective vaccination cohorts excluded due to history-reported or serologically detected (positive anti-nucleocapsid antibody) SARS-CoV-2 infection. |
| **eTable 3.** | Data acquisition by questionnaire. Reactogenicity within seven days after first/second and booster vaccination. |
| **eTable 4.** | Reported adverse drug reactions (ADR) after first, second, and booster vaccination. |
| **eTable 5.** | Severity of adverse drug reactions (ADR) after first, second, and booster vaccination. |
| **eTable 6.** | Incapacity to work**.** Rate of incapacity to work after first, second, and booster vaccination**.** |
| **eTable 7.** | Distribution of sex in the four study cohorts. |
| **eTable 8.** | Distribution of sex among the occupational groups of the hospital staff. |
| **eTable 9.** | Comparison of men and women with regard to anti-S RBD concentrations,  exemplary for two study visits. |
| **eFigure 1.** | Study Flowchart – Basic Immunization. |
| **eFigure 2.** | Study Flowchart – Booster Vaccination. |
| **eFigure 3.** | Effect of booster vaccination on anti-S RBD kinetics. |

**Study Cohorts**

The basic immunizations (first and second vaccination) were performed using the m-RNA vaccine BNT162b2 (BioNTech/Pfizer, Mainz, Germany; **B**) and the vector-based vaccine ChAdOx1-S (AstraZeneca, Wilmington, DE, USA; **A**).

| **Cohort** | **Homologous Vaccination Schedule** |
| --- | --- |
| **BB-3** | BNT162b2 - BNT162b2, Vaccination Interval 3 weeks |
| **BB-6** | BNT162b2 - BNT162b2, Vaccination Interval 6 weeks |
| **AA-12** | ChAdOx1-S - ChAdOx1-S, Vaccination Interval 12 weeks |
|  | **Heterologous Vaccination Schedule** |
| **AB-12** | ChAdOx1-S - BNT162b2, Vaccination Interval 12 weeks |

|  | | **anti-S RBD after Basic Vaccination** | | | **anti-S RBD before/after BNT162b2 Booster** | | |
| --- | --- | --- | --- | --- | --- | --- | --- |
|  |  | **after 4 weeks** | **after 3 months** | **after 6 months** | **before Boost** | **after 4 weeks** | **after 3 months** |
| **BB-3** | Median (U/mL) | 2,156.0 | 1,116.0 | 769.1 | 501.3 | 25,000.0 | 13,880.0 |
|  | 25th percentile (U/mL) | 1,188.0 | 725.9 | 509.1 | 326.0 | 18,473.8 | 8,755.2 |
|  | % > 2,500 U/mL * | 39.5 | 11.8 |  |  |  |  |
|  | % > 25,000 U/mL * | not available | not available | 0.0 | 0.0 | 58.9 | 20.3 |
| **BB-6** | Median (U/mL) | 2,500.0 | 3,104.0 | 1,188.0 | 926.8 | 18,670.0 | 8,649.5 |
|  | 25th percentile (U/mL) | 2,500.0 | 1,769.0 | 830.0 | 575.7 | 14,427.5 | 5,909.8 |
|  | % > 2,500 U/mL * | 91.9 | 65.5 |  |  |  |  |
|  | % > 25,000 U/mL * | not available | 0.0 | 9.4 | 0.0 | 26.5 | 5.0 |
| **AA-12** | Median (U/mL) | 1,112.0 | 517.8 | 271.8 | 267.7 | 16,488.0 | 8,685.0 |
|  | 25th percentile (U/mL) | 575.0 | 317.9 | 153.5 | 151.8 | 11,191.5 | 6,251.5 |
|  | % > 2,500 U/mL * | 15.1 | 4.8 |  |  |  |  |
|  | % > 25,000 U/mL * | 0.0 | 0.0 | 0.0 | 0.0 | 24.4 | 2.0 |
| **AB-12** | Median (U/mL) | 9,016.5 | 3,609.0 | 1,362.0 | 1,073.0 | 15,170.5 | 7,005.5 |
|  | 25th percentile (U/mL) | 5,678.2 | 2,186.2 | 841.8 | 639.5 | 10,393.0 | 4,269.0 |
|  | % > 2,500 U/mL * | 95.6 | 68.3 |  |  |  |  |
|  | % > 25,000 U/mL * | 4.7 | 2.5 | 0.4 | 0.0 | 20.6 | 3.6 |

**eTable 1.**

**Descriptive Statistics of anti-Spike Receptor Binding Domain (anti-S RBD) and Representation of Upper Limits of Quantification (ULoQ) at the different Study Visits.**

Due to changes in assay performance by the manufacturer, the ULoQ has changed during the study period from 2,500 U/mL to 25,000 U/mL.

* Proportion of Study Subjects with an anti-S RBD Concentration above the specified ULoQ.

| **Subjects excluded or not considered for analyses / respective population (%)** | | | | | | |
| --- | --- | --- | --- | --- | --- | --- |
|  | **BB-3** | | **BB-6** | **AA-12** | **AB-12** | **Total** |
| **Before end of basic immunization (n = 1,120)** | | | | | | |
|  | | 1* / 511  (0.20%) | | 1* / 609  (0.16%) | | 2* / 1,120  (0.18%) |
|  | | | | | | |
| **Study visits after basic immunization (n = 1,118)** | | | | | | |
| **Visit 1**  after 4 weeks | | 6 / 248  (2.42%) | 1 / 262  (0.38%) | 2 / 265  (0.75%) | 4 / 343  (1.17%) | 13 / 1,118  (1.16%) |
| **Visit 2**  after 3 months | | 1 / 242  (0.41%) |  |  |  | 1 / 1,105  (0.09%) |
| **Visit 3**  after 6 months | | 1 / 241  (0.41%) |  | 1 / 263  (0.38%) |  | 2 / 1,104  (0.18%) |
|  | | | | | | |
| **Study visits after BNT162b2 booster (n = 485)** | | | | | | |
| **Visit 4**  before Booster | |  |  |  |  | 0 / 485  (0.0%) |
| **Visit 5**  after 4 weeks | |  | 1 / 101  (0.99%) |  |  | 1 / 485  (0.21%) |
| **Visit 6**  after 3 months | | 1 / 130  (0.77%) |  | 1 / 96  (1.04%) | 1 / 158  (0.63%) | 3 / 484  (0.62%) |
| * Excluded subjects who reported previous SARS-CoV-2 infection during the second vaccination.  All other excluded subjects were detected by a positive anti-Nucleocapsid-antibody during study visits. | | | | | | |

**eTable 2.**

**Subjects who were excluded from the study due to a history of reported SARS-CoV-2 infection or subjects in the respective vaccination cohorts who were not considered for the subsequent immunogenicity analyses due to a serologically detected SARS-CoV-2 infection (positive anti-nucleocapsid antibodies).**

In addition, the proportion of excluded subjects in the respective population (n/N (%)) is shown.

| **First**  **Vaccination** | **BNT162b2**  478 were included in analysis  28 did not return questionnaire | | **ChAdOx1-S**  533 were included in analysis  80 did not return questionnaire | |
| --- | --- | --- | --- | --- |
| **Second**  **Vaccination** | **BNT162b2**  study cohort BB-3  216 were included in analysis  32 did not return questionnaire | **BNT162b2**  study cohort BB-6  195 were included in analysis  63 did not return questionnaire | **ChAdOx1-S**  study cohort AA-12  224 were included in analysis  43 did not return questionnaire | **BNT162b2**  study cohort AB-12  273 were included in analysis  73 did not return questionnaire |
| **Booster**  **Vaccination** | **BNT162b2**  study cohort BB-3  105 were included in analysis  41 did not return questionnaire | **BNT162b2**  study cohort BB-6  62 were included in analysis  43 did not return questionnaire | **BNT162b2**  study cohort AA-12  57 were included in analysis  42 did not return questionnaire | **BNT162b2**  study cohort AB-12  66 were included in analysis  97 did not return questionnaire |

**eTable 3.**

**Data acquisition by questionnaire. Reactogenicity within seven days after first/second and booster vaccination.**

|  | Participants, No. (%) | | | | |
| --- | --- | --- | --- | --- | --- |
| Study-Cohort | no ADR | only local ADR | only systemic ADR | local and systemic ADR | Total |
| after first vaccination | | | | | |
| BB-3 | 63 (26.7) | 75 (31.8) | 19 (8.0) | 79 (33.5) | 236 (100) |
| BB-6 | 63 (26.8) | 51 (21.7) | 28 (11.9) | 93 (39.6) | 235 (100) |
| AA-12 | 25 (10.4) | 9 (3.8) | 47 (19.5) | 160 (66.4) | 241 (100) |
| AB-12 | 14 (4.9) | 5 (1.7) | 55 (19.1) | 214 (74.3) | 288 (100) |
| BB-3 vs BB-6: ns; AA-12 vs AB-12: p=.036;  all other intergroup comparisons: p<0.001 | | | | | |
| after second vaccination | | | | | |
| BB-3 | 14 (6.6) | 25 (11.8) | 20 (9.5) | 152 (72.0) | 211 (100) |
| BB-6 | 33 (17.0) | 26 (13.4) | 20 (10.3) | 115 (59.3) | 194 (100) |
| AA-12 | 97 (44.1) | 40 (18.2) | 23 (10.5) | 60 (27.3) | 220 (100) |
| AB-12 | 35 (13.1) | 47 (17.5) | 24 (9.0) | 162 (60.4) | 268 (100) |
| BB-3 vs BB-6: p=.007; BB-3 vs AB-12: p=.021;  AB-12 vs BB-6: ns;  all other intergroup comparisons: p<0.001 | | | | | |
| after booster vaccination | | | | | |
| BB-3 | 2 (1.9) | 16 (15.2) | 10 (9.5) | 77 (73.3) | 105 (100) |
| BB-6 | 7 (11.3) | 18 (29.0) | 6 (9.7) | 31 (50.0) | 62 (100) |
| AA-12 | 4 (7.0) | 10 (17.5) | 3 (5.3) | 40 (70.2) | 57 (100) |
| AB-12 | 3 (4.5) | 18 (27.3) | 3 (4.5) | 42 (63.6) | 66 (100) |
| BNT-3 vs BNT-6: p=0.004;  all other intergroup comparisons: ns | | | | | |

**eTable 4.**

**Reported adverse drug reactions (ADR) after first, second, and booster vaccination.**

Statistical analysis was performed with Chi^2^-Test, ns = not significant.

|  | Participants, No. (%) | | | | |
| --- | --- | --- | --- | --- | --- |
| Study-Cohort | Mild,  no impairment of daily activities | Moderate, impairment of daily activities | Severe,  not possible to carry out daily activities | Presenting at emergency room | Total |
| after first vaccination | | | | | |
| BB-3 | 102 (68.0) | 47 (31.3) | 1 (0.7) | 0 (0) | 150 (100) |
| BB-6 | 99 (60.0) | 64 (38.8) | 2 (1.2) | 0 (0) | 165 (100) |
| AA-12 | 53 (24.7) | 96 (44.7) | 66 (30.7) | 0 (0) | 215 (100) |
| AB-12 | 46 (16.7) | 139 (50.5) | 89 (32.4) | 1 (0.4) | 275 (100) |
| BB-3 vs BB-6: ns; AA-12 vs AB-12: ns;  all other intergroup comparisons: p<0.001 | | | | | |
| after second vaccination | | | | | |
| BB-3 | 55 (27.8) | 106 (53.5) | 37 (18.7) | 0 (0) | 198 (100) |
| BB-6 | 67 (42.1) | 64 (40.3) | 28 (17.6) | 0 (0) | 159 (100) |
| AA-12 | 82 (65.6) | 29 (30.6) | 6 (4.8) | 0 (0) | 125 (100) |
| AB-12 | 114 (48.3) | 100 (42.4) | 22 (9.3) | 0 (0) | 236 (100) |
| BB-3 vs BB-6: p=0.013; BB-6 vs AB-12: p=0.049; AA-12 vs AB-12: p=0.006  all other intergroup comparisons: p<0.001 | | | | | |
| after booster vaccination | | | | | |
| BB-3 | 33 (32.0) | 49 (47.6) | 20 (19.4) | 1 (1.0) | 103 (100) |
| BB-6 | 38 (70.4) | 14 (25.9) | 2 (3.7) | 0 (0) | 54 (100) |
| AA-12 | 27 (50.9) | 23 (43.4) | 3 (5.7) | 0 (0) | 53 (100) |
| AB-12 | 42 (66.7) | 15 (23.8) | 6 (9.5) | 0 (0) | 63 (100) |
| BB-3 vs AA-12: p=0.039; AA-12 vs AB-12: ns;  BB-6 vs AA-12: ns; BB-6 vs AB-12: ns;  all other intergroup comparisons: p<0.001 | | | | | |

**eTable 5.**

**Severity of adverse drug reactions (ADR) after first, second, and booster vaccination.**

Statistical analysis was performed with Chi^2^-test, ns = not significant.

|  | Incapacity to work, n/N (%) | | |
| --- | --- | --- | --- |
| Study-Cohort | after first vaccination | after second vaccination | after booster vaccination |
| BB-3 | 4 / 449 (0.9) | 14 / 202 (6.9) | 2 / 114 (1.8) |
| BB-6 |  | 8 / 193 (4.1) | 1 / 65 (1.5) |
| AA-12 | 76 / 527 (14.4) | 3 / 217 (1.4) | 2 / 97 (2.1) |
| AB-12 |  | 9 / 268 (3.4) | 5 / 91 (5.5) |
| Total | 80 / 976 (8.2) | 34 / 880 (3.9) | 10 / 367 (2.7) |
|  |  |  |  |
|  | first vaccination with BNT162b2 (BB-3 and BB-6) vs first vaccination with ChAdOx1-S (AA-12 and AB-12): p<0.001 | BB-3 vs BB-6: p=0.023  BB-3 vs AA-12: p=0.003  BB-3 vs AB-12: p=0.021  AA-12 vs AB-12: ns  BB-6 vs AB-12: ns | all intergroup comparisons: ns |

**eTable 6.**

**Incapacity to work. Rate of incapacity to work after first, second, and booster vaccination.**

Numerical differences of the individual characteristics to the total population result from missing data in the questionnaire. Statistical analysis was performed with Chi^2^-test, ns = not significant.

In the German health care system and particularly hospitals, nursing care and medical assistant professions are usually performed by women rather than men. This is also reflected in our study with a female proportion of almost 3/4 of the total study cohort. In this respect, the female sex could represent a confounding factor with regard to the investigated antibody concentrations.

To this end, we conducted further investigations:

The first step was to investigate whether the sex distributions differed in the four study cohorts (eTable 7).

Similar sex distributions were found among the groups with the exception of group BB-3 having a statistically significant higher proportion of men compared to the other groups.

|  | | Sex Distribution | | | | |
| --- | --- | --- | --- | --- | --- | --- |
| Study Cohort | | **BB-3** | **BB-6** | **AA-12** | **AB-12** | **Total** |
|  | | | | | | |
| male | n/N | 93 / 245 | 50 / 257 | 59 / 260 | 55 / 328 | 257 / 1,090 |
|  | % | 38.0 | 19.5 | 22.7 | 16.8 | 23.6 |
| female | n/N | 152 / 245 | 207 / 257 | 201 / 260 | 273 / 328 | 833 / 1,090 |
|  | % | 62.0 | 80.5 | 77.3 | 83.2 | 76.4 |
| BB-3 vs BB-6, BB-3 vs AA-12 and BB-3 vs AB-12: each p<0.001  all other intergroup comparisons: each ns | | | | | | |

**eTable 7.**

**Distribution of sex in the four study cohorts.**

Numerical differences of the individual characteristics to the total population result from missing data in the questionnaire. Statistical analysis was performed with Chi^2^-test, ns = not significant.

The deviating sex distribution in only one cohort can be explained by the vaccination chronology of the study cohorts. At the start of the study, subjects with two BNT162b2 vaccinations at three-week intervals were initially included and thus assigned to group BB-3.

At the beginning of the national vaccination campaign, however, hospital staff with professions close to patients were initially considered for primary SARS-CoV-2 vaccination due to an increased infection risk.

In this respect, nurses (occupational group dominated by females at 83.6%) and physicians (49.2% female, and thus almost balanced sex distribution, see eTable 8) initially qualified.

Consequently, there was a higher proportion of men in the chronologically first vaccination cohort BB-3 than in all other cohorts.

|  | | | | | | |
| --- | --- | --- | --- | --- | --- | --- |
|  | | | **Occupational Group** | | | **Total** |
|  |  |  | **Nursing** | **Physicians** | **Others*** |  |
| **Sex** | **male** | N | 68 | 92 | 88 | 248 |
|  |  | % within Sex | 27.4 | 37.1 | 35.5 | 100.0 |
|  |  | % within Occupational Group | 16.4 | 50.8 | 18.9 | 23.4 |
|  | **female** | N | 347 | 89 | 378 | 814 |
|  |  | % within Sex | 42.6 | 10.9 | 46.4 | 100.0 |
|  |  | % within Occupational Group | 83.6 | 49.2 | 81.1 | 76.6 |
| **Total** | | N | 415 | 181 | 466 | 1,062 |
|  |  | % within Sex | 39.1 | 17.0 | 43.9 | 100.0 |
|  |  | % within Occupational Group | 100.0 | 100.0 | 100.0 | 100.0 |

**eTable 8. Distribution of sex among the occupational groups of the hospital staff.**

*Other hospital professions.

Numerical differences between individual characteristics and the total population are due to missing information in the questionnaire.

In a second step, the antibody concentrations (anti-S RBD, U/mL) of women were compared with those of men at the same study visits within a cohort.

Two study visits with a low proportion of subjects with antibody levels above the upper limit of quantification (ULoQ) were selected as examples (eTable 9).

Women had significantly higher antibody concentrations (anti-S RBD) than men in only two of a total of eight sex-specific comparative analyses (eTable 9).

In summary, given the sex distribution within the four study cohorts on the one hand and given the inconsistently higher antibody concentrations in women on the other hand, the "sex factor" can be considered subordinate in our analyses.

In this respect, the key statements of the present work are not fundamentally affected.

| **Study Visit**  **anti-Spike RBD (U/mL)** | |  | **BB-3** | | **BB-6** | | **AA-12** | | **AB-12** | |
| --- | --- | --- | --- | --- | --- | --- | --- | --- | --- | --- |
|  |  |  | **male**  **(N=93)** | **female**  **(N=152)** | **male**  **(N=50)** | **female**  **(N=207)** | **male**  **(N=59)** | **female**  **(N=201)** | **male**  **(N=55)** | **female**  **(N=273)** |
| **Six months**  **after basic immunization** | Min / Max | | 68.3 / 3,943.0 | 113.6 / 6,740.0 | 142.3 / 5,451.0 | 198.4 / 5,870.0 | 25.5 / 1,373.0 | 4.2 / 3,390.0 | 423.3 / 5,415.0 | 171.8 / 25,000.0 |
|  | Median [IQR] | | 764.3 [518.5;1,341.0] | 864.0 [505.5;1,286.5] | 747.1 [418.0;1,272.0] | 1,262.0 [882.0;1,803.0] | 241.9 [137.6;359.9] | 278.9 [156.8;532.4] | 1,494.0 [736.7;2,248.5] | 1,349.0 [856.1;2,039.0] |
|  | Mean (std) | | 1,023.5 (780.4) | 1,026.3 (822.7) | 1,283.4 (1,565.4) | 1,456.9 (921.3) | 355.7 (362.2) | 452.3 (529.0) | 1,740.5 (1,151.0) | 1,954.1 (2,460.6) |
|  | N (NA) | | 57 (36) | 111 (41) | 12 (38) | 115 (92) | 30 (29) | 150 (51) | 36 (19) | 209 (64) |
| **Three months**  **after boost** | Min / Max | | 6,307.0 / 25,000.0 | 2,529.0 / 25,000.0 | 1,030.0 / 25,000.0 | 2,778.0 / 25,000.0 | 2,603.0 / 9,964.0 | 3,675.0 / 25,000.0 | 2,351.0 / 25,000.0 | 1,342.0 / 25,000.0 |
|  | Median [IQR] | | 13,970.0 [8,032.0;25,000.0] | 13,836.0 [8,926.0;20,922.0] | 8,122.0 [5,069.5;17,191.8] | 8,790.5 [6,010.2;12,255.2] | 6,629.0 [4,319.8;8,908.2] | 8,984.5 [6,686.5;13,633.8] | 7,040.0 [4,114.5;18,322.5] | 6,971.0 [4,281.0;11,158.0] |
|  | Mean (std) | | 15,480.5 (7,758.1) | 14,655.0 (6,849.8) | 11,090.2 (9,430.0) | 10,087.9 (5,535.4) | 6,480.4 (2,905.8) | 10,196.6 (4,909.5) | 10,780.5 (8,899.7) | 8,510.9 (5,646.1) |
|  | N (NA) | | 17 (76) | 57 (95) | 6 (44) | 54 (153) | 8 (51) | 42 (159) | 11 (44) | 73 (200) |
| BB-6 at six months after basic immunization: p=0.037; AA-12 at three months after boost: p=0.041,  all other comparisons (male vs. female): ns; statistical analysis was performed with Wilcoxon-test, ns = not significant.  N: number of subjects in follow-up with antibody measurement; NA: not available due to loss to follow-up | | | | | | | | | | |

**eTable 9.**

**Comparison of men and women with regard to anti-S RBD concentrations, exemplary for two study visits.**

**eFigure 1.**

**Study Flowchart – Basic Immunization.**

**eFigure 2.**

**Study Flowchart – Booster Vaccination.**

Detection of the boost effect is based on the anti-S RBD concentrations of the study subjects. However, some of these measured values were above the Upper Limit of Quantification (ULoQ). In addition to the presentations in the main article, identical analyses are presented below with only continuous antibody concentrations (anti-S RBD) within the measurement range of the test (eFigure 3):

The distribution of boost factors within the four vaccination groups differs only in the intergroup comparison BB-3 vs AA-12 (not significant) compared to the analysis in the main article (p=.037; [Wilcoxon test]). This result is irrelevant for the further analysis (Panel B), as the AA-12 cohort is not considered further anyway due to the aspects described in the main article.

The Spearman correlation shown in Panel B (r=0.51; 95% CI 0.38-0.62; p<0.001), taking into account only continuous antibody values, gives almost identical results to the main article (r=0.51; 95% CI 0.41-0.6; p<0.001).

The key statements of the main article are therefore not relevantly affected by measured values above the ULoQ.


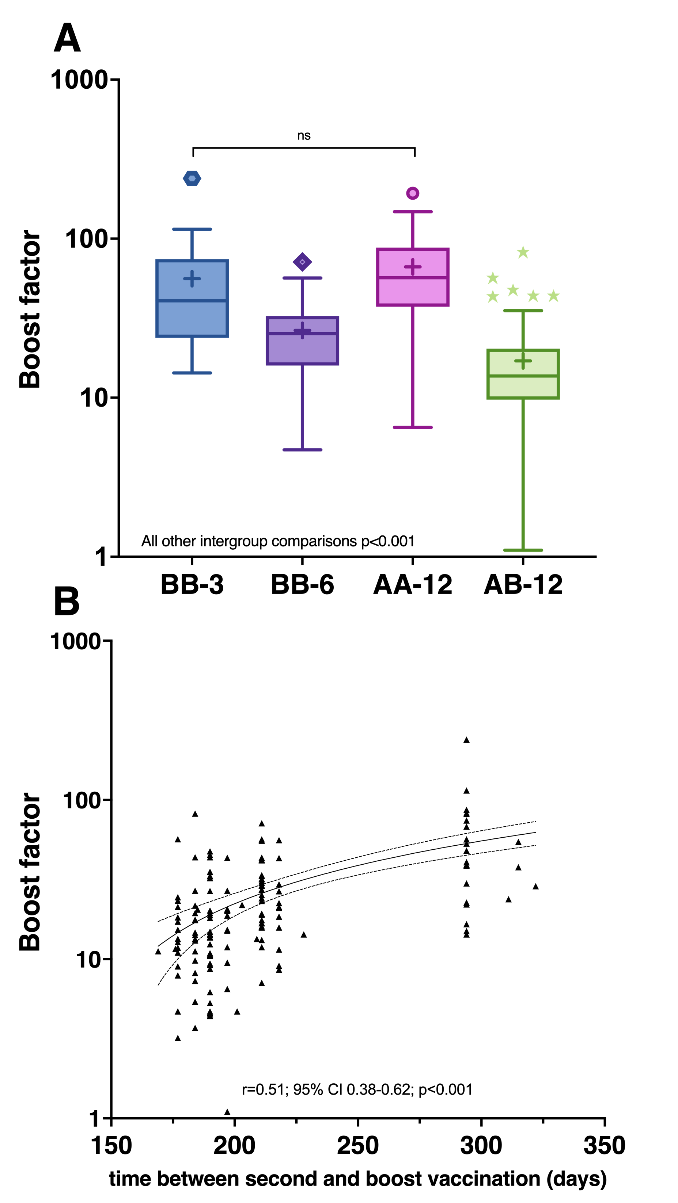


**eFigure 3.**

**Effect of booster vaccination on anti-S RBD kinetics.**

Boost-factor: quotient of anti-S RBD levels four weeks after and levels immediately before the booster vaccination; only continuous anti-S RBD concentrations were considered.

**Panel A**: Booster effect per vaccination-cohort. Data are presented in the standard box plot format: The box ranges from the first quartile (Q1) to the third quartile (Q3) of the distribution and the range represents the interquartile range (IQR). The median is represented by a line across the box, the mean is depicted by a "+" in the box. Whiskers range from Q1 and Q3 to 1.5 times IQR. Data beyond the whiskers are shown as outliers. The statistical analysis was performed using the Wilcoxon test.

**Panel B**: Correlation between boost factor and time-interval (in days) between second and booster vaccination, without considering the homologous ChAdOx1-S vaccination group, which was cross-vaccinated with m-RNA vaccine for the first time. Each symbol represents an individual subject. As a statistical test, a Spearman Correlation was used.
